# The Cyclic Adenosine Monophosphate Phosphodiesterase CpdA Modulates cAMP Homeostasis, Stress Adaptability, Biofilm Formation, Motility, Quorum Sensing and Antibiotic Resistance of *Aeromonas veronii*

**DOI:** 10.4014/jmb.2511.11034

**Published:** 2026-04-15

**Authors:** Wenjing Yang, Xiaolei Wang, Shengyuan Zhou, Guina Yu, Xiaoyan Zhou, Zhihai Sui

**Affiliations:** School of Life Science, Linyi University, Linyi 276000, P. R. China

**Keywords:** *Aeromonas veronii*, cAMP phosphodiesterase, Adversity tolerance, Biofilm formation, Motility, Antibiotic resistance

## Abstract

*Aeromonas veronii*, a Gram-negative pathogen, is ubiquitous in nature and infects broad hosts, which becomes a serious threat to the public health. The cyclic adenosine monophosphate (cAMP), precisely modulated by cyclic nucleotide phosphodiesterase (PDE), regulates key cellular processes in bacteria. However, the specific function and regulatory role of PDE in *A. veronii* remain unclear. In this study, a Class III PDE gene named as *cpdA* in *A. veronii* WL-3 was identified for the first time, which shared 39.18%-49.19% identity with homologs from other bacteria and also contained the conserved metal-binding motif. For functional elucidation, a *cpdA*-deleted mutant WL-3Δ*cpdA* was constructed. The results showed that the intracellular cAMP level in WL-3Δ*cpdA* was 2.34-fold higher than that in WL-3, confirming the PDE activity *in vivo*. WL-3Δ*cpdA* exhibited impaired growth under the normal condition and reduced tolerance to NaCl and pH stresses. Furthermore, the deficiency of *cpdA* significantly inhibited biofilm formation and swimming motility, which were consistent with aberrant flagellar structure and decreased N-acyl-homoserine lactone production. In contrast, the expression of nine flagella-related genes and two quorum-sensing genes was upregulated. Interestingly, WL-3Δ*cpdA* conferred higher resistance to partial β-lactam antibiotics with upregulation of *bla*_CphA3_ and *bla*_OXA-12_, but heightened sensitivity to kanamycin, neomycin, polymyxin B, and erythromycin, positioning CpdA as a multifaceted effector. Genetic complementation partially or fully reversed the most defects of WL-3Δ*cpdA*. These results identify that CpdA is involved in regulating various physiological properties of *A. veronii*, thus nominating CpdA as an attractive and novel target for anti-infective strategies.

## Introduction

In bacteria, cyclic 3',5'-adenosine monophosphate (cAMP), as an important second messenger, is first discovered in *Escherichia coli* in 1965 and plays a vital role in response to extracellular environment change [1, 2]. cAMP regulates the expression of genes involved in various cellular processes, such as carbon metabolism, SOS response and virulence, by the direct binding with cAMP-dependent protein kinase (PKA) and cAMP-receptor protein (CRP) [3-5]. The intracellular homeostasis of cAMP is mainly maintained through a balance between the production from ATP via adenylate cyclase (AC) and the degradation to 5'-AMP by cyclic nucleotide phosphodiesterase (PDE) [6].

Members of the PDE superfamily are divided into three classes (I, II, and III) based on sequence homology [7]. Among three classes, Class III PDEs are widely distributed in most bacteria, in contrast to the more restricted presence of Class I and II PDEs, indicating that Class III PDEs play a fundamental and conserved physiological role in bacterial cAMP homeostasis [8-10]. Class III PDEs, such as CpdA from *Escherichia coli* and Rv0805 from *Mycobacterium tuberculosis*, are characterized by the conserved sequence motif of D-[X]n-GD-[X]n-GNH[E/D]-[X]n-H-[X]n-GHXH, which clusters to form the metal coordination center for the enzyme activity [11-13]. Metal ions (*e.g*., Fe^3+^, Mn^2+^, Mg^2+^) are required for the activation and dimerization of Class III PDEs [14]. In addition to the control of cAMP level, PDEs also modulate the physiological functions and pathogenicity in bacteria [7, 15]. For example, the Class III PDE CpdS from *Serratia marcescens* mediates biofilm formation through type I fimbriae [16]. Besides, the Class II PDE Rv1339 in *M. smegmatis* disrupts the biosynthesis of peptidoglycan and reduced tolerance to cell wall–targeting antimicrobials [17].

*Aeromonas veronii*, belonging to the genus *Aeromonas* within the family Aeromonadaceae, is an important Gram-negative pathogen and ubiquitous in nature with strong environmental adaptability [18, 19]. *A. veronii* infects a wide range of host, including fish, amphibians, birds and humans, which causes the septicemia, ulcerative syndrome, diarrhea, and even death [20-22]. For example, in aquaculture, *A. veronii* is a common pathogen and causes mass mortality in various fish species, with reported mortality rates of 40–60% in farmed Nile tilapia (*Oreochromis niloticus*) in India and 40–100% in channel catfish (*Ictalurus punctatus*) in Vietnam [23, 24] In addition, foods containing meat, milk and vegetables are contaminated by *A. veronii*, which poses a dreadful threat to food safety and storage [25].

For the control of bacterial infections, antibiotics remain the primary prophylactic and therapeutic agents, but their inappropriate use results in the dual challenges of drug residue accumulation and multi-drug resistance [26]. For example, *A. veronii* strains isolated from bluespot mullet (*Mugil seheli*, a commercially important Mugilidae fish widely distributed in the Indo-Pacific) carry *bla*_TEM_, *bla*_CTX-M_, *bla*_SHV_, *tetA*, *aadA1*, and *sul1* resistance genes, conferring resistance to several antimicrobial classes [27]. Similarly, human-derived *A. veronii* strains displays the multi-drug resistance to antibiotics including spectinomycin, gentamicin, streptomycin, and kanamycin, which causes difficulties in the treatment [28]. Therefore, developing alternative control strategies such as vaccines, phage therapy and enzyme inhibitors is imperative to combat the side effects of antibiotics [29]. Drugs targeting virulence-related factors such as quorum sensing (QS), biofilm, toxins and secretion systems disarm infectious pathogens without killing or strong selective pressure [30, 31].

The physiological functions of *A. veronii* are regulated by the signal molecules such as autoinducer-2 (AI-2), cyclic di-guanosine monophosphate (c-di-GMP) and N-acyl-homoserine lactones (AHLs) to facilitate adaptation to diverse environmental stresses and infection [32, 33]. For example, the sensory histidine kinase gene cheA regulated by AHLs-mediated QS promotes the intestinal colonization of *A. veronii* [34]. Although the *A. veronii* exhibits the second messenger cAMP like other bacteria, the regulatory mechanisms of its cAMP signaling pathway remain poorly characterized.

In this study, we identified and analyzed the sequence of *cpdA* gene encoding Class III PDE in *A. veronii*. Given the broad environmental distribution and robust stress adaptability of *A. veronii*, we hypothesized that CpdA proteinwould serve as a key regulator in modulating physiological responses. To verify this hypothesis, we constructed a *cpdA* knockout mutant, and investigated the biological effects on the cAMP hydrolysis, growth, stress tolerance, biofilm formation, motility, QS and antibiotic resistance. The results provide an experimental basis and insights for understanding the adaptation of *A. veronii* to the environment, laying a theoretical foundation for devising effective control measures.

## Materials and Methods

### Bacterial Strains and Growth Conditions

*A. veronii* WL-3 was isolated from the diseased northern snakehead (*Channa argus*) with infection symptoms of skin ulcers, ascites, and visceral hemorrhage. In addition, our previous study showed that *A. veronii* WL-3 harbored four virulence genes (*aer*, *alt*, *ahyB* and *act*) with optimal growth temperature of 37°C and pH of 7.0, which was similar to other *A. veronii* strains [35, 36]. The strain was cultured in Luria-Bertani (LB) medium (10 g/L tryptone, 5 g/L yeast extract, 10 g/L NaCl, pH 7.0) (Oxoid, UK) supplemented with ampicillin (Solarbio, China) at a final concentration of 15 μg/ml at 37°C. Before experiments, the strain was streaked on the LB agar plates twice for activation and then inoculated in the LB liquid medium. *E. coli* DH5α and S17-1λpir competent cells were purchased from Weidibio (China). *Chromobacterium violaceum* CV026 was purchased from BIOSCI (China). *E. coli* and *C. violaceum* were incubated in LB medium at 37°C and 30?, respectively. To screen for positive transformants, chloramphenicol (Solarbio) (30 μg/ml) and ampicillin (15 μg/ml) were added into LB medium as required.

### Sequence Analysis of CpdA in *A. veronii*

The predicted PDE gene in the genome of *A. veronii* WL-3 (GenBank No. GCA055557395.1), named as *cpdA* (GenBank No. PX935986), was identified through NCBI tBLASTn program (http://www.ncbi.nlm.nih.gov/blast/Blast.cgi) using the known CpdA sequence of *E. coli* (GenBank No. BAA03989) as a query. The distribution and identity of CpdA within *A. veronii* strains were investigated by using NCBI BLASTp program. The physicochemical properties of CpdA were analyzed using Expasy (https://web.expasy.org/protparam/). The amino acid sequence of CpdA was aligned with those of *Edwardsiella piscicida* (GenBank No. AGH72296), *E. coli* (GenBank No. BAA03989), *Vibrio vulnificus* (GenBank No. AAO65602), *Haemophilus influenzae* (GenBank No. HI0399), and *Pseudomonas aeruginosa* (GenBank No. PA4969) by DNAMAN 9.0 software (Lynnon Biosoft, Canada) [37]. For precise taxonomic classification, the phylogenetic analysis of the amino acid sequences from Classes I, II and III PDEs was performed via MEGA 12.0 software (USA) [38] with Neighbor-Joining (NJ) and Maximum Likelihood (ML) methods with 1000 bootstrap iterations.

### Construction of a *cpdA* Full-deletion Mutant and Complementary Strain

To create a full deletion mutant, the full-length *cpdA* gene (873bp), encoding a 290-amino-acid protein, was deleted from the genome of *A. veronii* WL-3 using the suicide plasmid-based method, as described previously [39]. In brief, the genomic DNA of *A. veronii* WL-3 was extracted by TIANamp Bacteria DNA Kit (Tiangen Biotech, China) as a template. Then, the upstream and downstream homologous arms of the *cpdA* gene were amplified by PCR with primers *cpdA*-up-F/R and *cpdA*-down-F/R, respectively. These amplified fragments were fused by overlap PCR using primers *cpdA*-up-F and *cpdA*-down-R to generate a fusion product. After the fusion fragment was purified using an E.Z.N.A.^®^ Gel Extraction kit (Omega Bio-Tek, Norcross, USA) and digested with *Bam*H I (TaKaRa, China), PCR product was ligated into the *Bgl* II (TaKaRa)-digested suicide vector pDM4 [40], which carries R6K *ori*, *sacB* sucrose-sensitivity gene, and chloramphenicol resistance gene. The resulting recombinant plasmid pDM4-Δ*cpdA* was introduced into *E. coli* S17-1λpir via heat shock transformation and subsequently transferred to *A. veronii* WL-3 through conjugation. Then, the single-crossover transconjugant was confirmed by PCR with primers *cpdA*-up-F/*cpdA*-down-R and positive clones were sub-cultured in fresh LB medium six times to induce a second single crossover. Double-crossover clones were selected on LB agar plates containing 12% sucrose (SCR, China) and confirmed via PCR with primers *cpdA*-F/R or *cpdA*-test-F/R, generating the *cpdA* knockout *A. veronii* strain WL-3Δ*cpdA*.

To create a complementary strain, as described previously [41], a DNA fragment (approximately 1370 bp) harboring the *cpdA* gene and its native promoter was amplified by PCR with primers *cpdA*-pBBR1MCS-F/R and cloned into the *Bam*H I-digested broad-host-range vector pBBR1-MCS. The recombinant plasmid pBBR1-MCS-*cpdA* was introduced into *E. coli* S17-1λpir and then conjugated into WL-3Δ*cpdA*, as described above. After selection with chloramphenicol and ampicillin, the positive colonies were identified by PCR with M13-F/R, yielding the complementary strain WL-3Δ*cpdA*C. The primers were synthesized by Tsingke Biotechnology Co., Ltd. (China), and the primer sequences are shown in Table 1.

### Intracellular cAMP Concentration Assay

*A. veronii* WL-3, WL-3Δ*cpdA* and WL-3Δ*cpdA*C were grown overnight, then sub-cultured into fresh LB medium at a 1:100 dilution and incubated to an optical density at 600 nm (OD_600_) of 1.0. Bacterial cells were harvested by centrifugation at 10,000 × g for 5 min at 4°C and resuspended in ice-cold sample dilution buffer (1 × PBS + 1% bovine serum albumin (BSA) + 0.5% Tween 20 + 0.04% Proclin 300) (Solarbio). After samples were lysed by sonication, the lysates were centrifuged at 10,000 × g for 10 min at 4°C to collect the supernatants. Then, the total protein concentration was determined using a BCA Protein Assay Kit (Solarbio) and the intracellular cyclic adenosine monophosphate (cAMP) content was quantified using a cAMP ELISA Kit (Elabscience, China). The intracellular cAMP level in *A. veronii* was obtained by calculating cAMP concentration per mg of total protein.

### Growth Curve Assays

*A. veronii* WL-3, WL-3Δ*cpdA* and WL-3Δ*cpdA*C were cultured overnight, then sub-cultured with 1:1000 ratio in 4 ml fresh LB medium and grown with shaking at 37°C to OD_600_ of 0.5. Subsequently, the cultures were diluted 1:100 into 100 ml of the appropriate LB liquid medium, which were prepared as follows for each stress condition. For pH stress tests, the pH of the liquid LB medium was adjusted to 5, 6, 8 and 9 with hydrochloric acid (HCl) (Kaier Chemical Co. Ltd., China) or sodium hydroxide (NaOH) (Tianjin Rgent Chemicals Co. Ltd., China), respectively. For osmotic stress tests, the LB medium contained 0%, 0.5%, 2%, and 4% (w/v) of NaCl, respectively. Then, the bacteria were incubated at 37°C for 24 h and OD_600_ was measured at 2 h intervals with an ultraviolet spectrophotometer (UV-9000S, Metash Instruments, China) to monitor the growth kinetics.

### Biofilm Formation Assays

*A. veronii* WL-3, WL-3Δ*cpdA* and WL-3Δ*cpdA*C were cultured to an OD_600_ of 0.5 and diluted at 1:1000 in LB liquid medium. 200 μl of the above dilution was dispensed into a polystyrene 96-well microplate (Corning, USA) and incubated at 37°C for 12 h. After static incubation, the culture media were aspirated and the adherent cells were washed three times with phosphate buffered saline (PBS) (Procell, China). After air-drying, 200 μl Bouin's fixative (Macklin, China) was added to each well and incubated in the dark for 2 h for cell fixation. After the wells were washed three times with 200 μl of PBS and air-dried at room temperature, the cells were stained with 200 μl of 1% crystal violet solution (Solarbio) for 30 min. Following the above washing and drying steps, the bound crystal violet was solubilized with 200 μl of 95% methanol (YONGDA, China) for 10 min and OD_570_ was measured using a microplate reader (Synergy 2; BioTek, USA).

### Motility Assays

As described previously [42], swimming and swarming motilities of *A. veronii* were assessed on soft agar plates containing 0.3% and 0.6% agar (BioFroxx, China), respectively. *A. veronii* WL-3, WL-3Δ*cpdA* and WL-3Δ*cpdA*C were pre-cultured to OD_600_ of 0.5 in LB medium at 37°C. 1 μl aliquot of the standardized bacterial suspension was spotted onto the center of LB plates containing 0.3% or 0.6 % (w/v) agar and the plates were incubated at 37°C for 24 h in the upright position. The diameters of the bacterial halos from the edge of the inoculation spot to the outermost edge of growth were measured to assess the motility differences among the strains. All measurements were performed in at least triplicate.

### The Observation of Flagellar Morphology

*A. veronii* WL-3, WL-3Δ*cpdA* and WL-3Δ*cpdA*C were cultured in liquid LB medium to the logarithmic growth phase (OD_600_ of 0.6). Cells were harvested by centrifugation at 10,000 × g for 1 min and the resulting pellets were resuspended in PBS. Then, the bacterial samples were adsorbed onto Formvar/carbon-coated copper grids (Beam Convergence Technology Co., Ltd., China) for 1 min and negatively stained with 2% phosphotungstic acid (pH 7.0) (Rhawn, China) for 1 min. The morphological changes of flagella were observed with a HT7800 transmission electron microscope (TEM) (Hitachi, Japan). The percentage of cells with intact or damaged flagella was assessed by analyzing the TEM images (n > 100 cells).

### The Detection of AHL Production

*C. violaceum* CV026 was used as reporter bacteria for the AHL production in QS by quantifying violacein synthesis [43]. The *C. violaceum*

CV026 was cultured in LB liquid medium at 30°C until the OD_600_ reached 0.5. After 1 ml of cultures was centrifugated at 5000 × g for 2 min, the bacterial pellet was resuspended in 1 ml of PBS. Then, 10 ml of the bacterial suspension was thoroughly mixed with 100 ml of molten LB agar medium, and poured into plates. *A. veronii* WL-3, WL-3Δ*cpdA* and WL-3Δ*cpdA*C were cultured to OD_600_ of 0.5 at 37°C and centrifuged at 10,000 × g for 10 min at 4°C. Then, 50 μl of the above supernatants was added to wells punched into the plates, respectively. The supernatant of *C. violaceum* CV026 was added as negative control and 10 μmol/ml N-hexanoyl-homoserine lactone (C_6_-HSL) (Sigma-Aldrich, USA) was added as positive control. After incubation at 30°C for 24 h, violacein production was quantified by measuring the diameters of violet halos surrounding the wells to evaluate the ability to produce AHL.

### Quantitative Real-Time Polymerase Chain Reaction (qRT-PCR) Assay

The total RNAs of *A. veronii* WL-3, WL-3Δ*cpdA* and WL-3Δ*cpdA*C were extracted with Trizol reagent (SimGen, China) and incubated with DNase I (Omega Bio-Tek, Norcross, USA) at 37°C for 5 min. The quality and quantity of total RNA were determined by electrophoresis and spectroscopic measurements with NanoDrop^TM^ One (ThermoFisher, UK). Then, the 1 μg RNA was reverse-transcribed into cDNA using the reverse transcription reaction kit (Toyobo, Japan). The qRT-PCR reaction was carried out by using the TB Green Premix Ex Taq^TM^ II FAST qPCR (Takara, China). All primers for the *cpdA* gene, flagellum-related genes (*fliE*, *flhA*, *flhB*, *fliD*, *flgK*, *flgB*, *flgL*, *flgE*, and *flgC*), QS-associated genes (*luxI* and *luxR*), drug resistance-related genes (*bla*_OXA-12_ and *bla*_CphA3_) and *crp* gene used in this study are listed in Table 2, with 16S rRNA as the internal reference. The qPCR program consisted of pre-denaturation at 95°C for 30 s, followed by 40 cycles of two-step amplification (95°C for 5 s and 60°C for 10 s), and a dissociation stage for melting curve (95°C for 15 s, 60°C for 30 s, and 95°C for 15 s). Then, the specificity of primers was assessed by melting curve analysis and 2% agarose (Tsingke, China) gel electrophoresis. In addition, the amplification efficiency of the primers was evaluated by using five-fold serial dilution of cDNA as templates for qRT-PCR and generating a standard curve by the ABI StepOne^TM^ system (ThermoFisher). Then, the amplification efficiency was calculated using the following formula: (10^–1/slope^–1) × 100%. After the validation of the specificity and high-efficiency (90%-110%) of primers (Fig. S1 and Table 2), wild-type WL-3 was used as a control and the expression levels of genes were calculated by 2^-ΔΔCT^ method [44].

### Antibiotic Susceptibility Test

The antibiotic susceptibility was determined with both Kirby Bauer disc diffusion method and broth microdilution method, respectively. For Kirby Bauer method, *A. veronii* WL-3 and WL-3Δ*cpdA* were grown to OD_600_ of 0.5 at 37°C. After washing twice with PBS, 100 μl of the bacterial suspension was spread on Mueller-Hinton (MH) agar plates (Oxoid). Then, the antimicrobial discs (BKMAM, China) (the name and concentration of antibiotic listed in Table 3) were placed on the plates and incubated at 37°C for 24 h. The diameter of the inhibition zones was measured to determine the change of antibiotic susceptibility.

Based on the above results, the minimum inhibitory concentration (MIC) of some antibiotics against *A. veronii* WL-3 and WL-3Δ*cpdA* were determined. *A. veronii* WL-3 and WL-3Δ*cpdA* were grown to OD_600_ of 0.5 and diluted to the 0.5 McFarland standard with MH medium. 4 μl of bacterial suspensions were inoculated into 200 μl of MH medium containing two-fold serially diluted antibiotics (from 512 μg/ml to 0.25 μg/ml) in a 96-well plate. The plates were incubated at 37°C for overnight and bacterial growth was assessed by measuring the OD_600_ using a microplate reader (Synergy 2, BioTek, USA). MIC was recorded as the lowest antibiotic concentration without visible bacterial turbidity. All MIC experiments were performed in triplicate.

### Statistical Analysis

All experiments in this study were performed at least three times. The data were collected and analyzed with GraphPad Prism 8 software (GraphPad Software, USA) by the analysis of variance (ANOVA). The data were expressed as the mean ± standard error of the mean (SEM) (N=3). Statistical significance was defined as * *p* < 0.05 and ** *p* < 0.01.

## Results

### The Characteristic of Putative Class III PDE CpdA in *A. veronii*

**Fig. 1:**
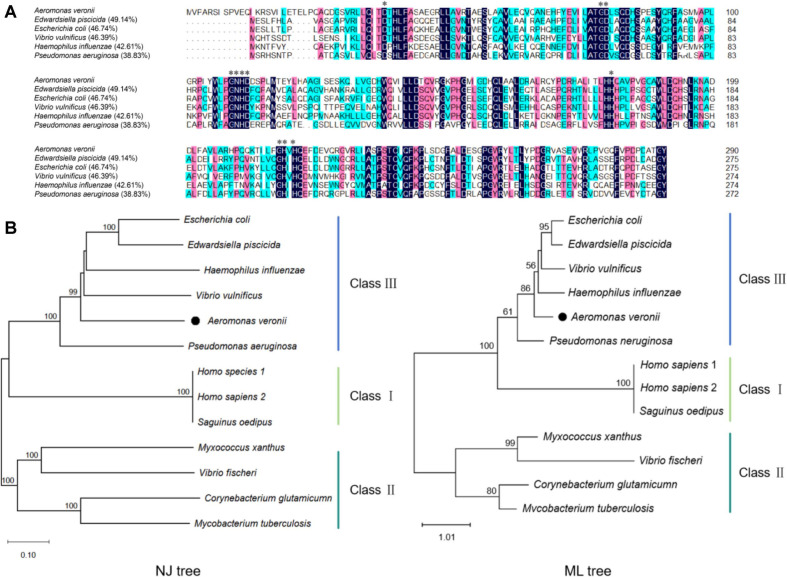
Bioinformatics analysis of CpdA homologues. (**A**) Multiple sequence alignment. Alignment of the sequences of the Class III CpdA family was performed with DNAMAN software. The consensus residues were marked in blue and residues with over 75% sequence identity were highlighted in pink. The star (*) indicates the conserved sequence motif. The sequence identity values between CpdA and other homologs are shown in bracket. (**B**) Phylogenetic analysis. The phylogenetic tree was generated by the Neighbor-joining (NJ) (left) and Maximum Likelihood (ML) (right) methods. The numbers above branches represent bootstrap support values derived from 1000 replicates. Only bootstrap values exceeding 50% are displayed.

tBLASTn analysis identified a putative Class III PDE in *A. veronii*, designated as CpdA, which consisted of 290 amino acid residues. The Expasy predicted that CpdA had a molecular weight of 32.29 kDa and an isoelectric point (pI) of 5.61. BLASTp showed that the putative *cpdA* gene was widely distributed in 90 *A. veronii* strains from NCBI database and exhibited high sequence identities (85.47% to 99.54%) among them. Multiple sequence alignment revealed that the animo acid sequence of CpdA in *A. veronii* shared identity of 49.14%, 46.74%, 46.39%, 42.61%, and 38.83% with that of *E. piscicida*, *E. coli*, *V. vulnificus*, *H. influenzae*, and *P. aeruginosa*, respectively (Fig. 1A). Furthermore, like other members of Class III PDE, the CpdA of *A. veronii* also contained the conserved sequence motif D-(X)n-GD-(X)n-GNH(E/D)-(X)n-H-(X)n-GHXH (Fig. 1A, star-labeled), which was critical for the Class III PDE activity. In addition, phylogenetic analysis showed that the CpdA of *A. veronii* clustered with the other Class III PDEs, not with Class I/II PDEs in NJ and ML trees (Fig. 1B). These results confirm the widespread presence of a Class III PDE homolog in *A. veronii*.

### Construction of the *cpdA* Deletion Mutant and Complementary Strain

**Fig. 2:**
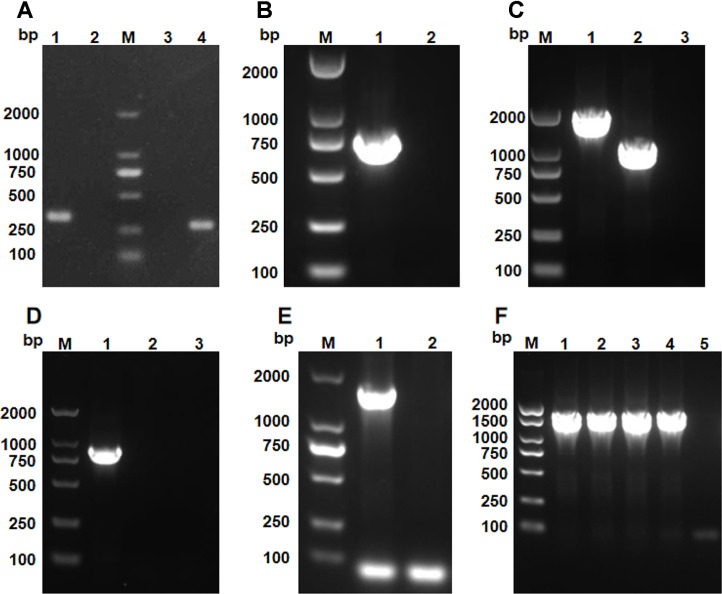
Construction of the *cpdA* deletion mutant and complementary strain. (**A**) Lane 1 and 4: PCR amplification of upstream and downstream homologous arms of the *cpdA* gene. Lane 2 and 3: PCR without template as negative control. (**B**) Lane 1: overlap PCR to fuse the upstream and downstream homologous arms. Lane 2: PCR without template as negative control. (**C**) The verification of double-crossover recombination by PCR with *cpdA*-test-F/R. Lane 1: PCR with WL-3 genome as positive control, Lane 2: PCR with WL-3Δ*cpdA* genome, Lane 3: PCR without template as negative control. (**D**) The detection of *cpdA* gene. Lane 1: PCR with WL-3 genome as positive control, Lane 2: PCR with WL-3Δ*cpdA* genome, Lane 3: PCR without template as negative control. (**E**) Lane 1: PCR to amplify the fragment containing *cpdA* and the promoter, Lane 2: PCR without template as negative control. (**F**) Lanes 1-4: PCR to identify the positive transformant clones; Lane 5: PCR without template as negative control.

To investigate the biological function of CpdA, the *cpdA* gene deletion mutant of *A. veronii* WL-3 was constructed using suicide plasmid-mediated homologous recombination. The 395 bp upstream and 358 bp downstream homologous arms, designed for *cpdA* deletion, were successfully amplified by PCR (Fig. 2A). Then, the 753 bp fusion fragment containing the upstream and downstream arms was created through overlap PCR (Fig. 2B). After double-crossover recombination and sacB-mediated sucrose counterselection, PCR using *cpdA*-test-F/R primers yielded a 985 bp product for the deletion strain, while the wild-type strain maintained the original 1858 bp fragment (Fig. 2C). We also found that when amplifying the *cpdA* gene with primers *cpdA*-F/R, no amplification product was detected in the deletion mutant whereas wild-type strain produced the expected band of *cpdA* gene (Fig. 2D). These results confirmed the successful construction of the *cpdA* deletion mutant, named as *A. veronii* WL-3Δ*cpdA*.

Also, the complementary strain was constructed using the vector pBBR1-MCS. The DNA fragment containing the *cpdA* gene and its promoter were amplified successfully as expected (Fig. 2E). The recombinant plasmid pBBR1-MCS-*cpdA* was then conjugated into the mutant strain WL-3Δ*cpdA*. The positive transformants have been verified by PCR with M13-F/R primers (Fig. 2F), generating the complementary strain WL-3Δ*cpdA*C.

To further confirm the successful construction of the strains WL-3Δ*cpdA* and WL-3Δ*cpdA*C, the expression level of *cpdA* was detected by qRT-PCR. The result showed that the expression of *cpdA* was detected in complementary strain WL-3Δ*cpdA*C, although it was 0.27-fold lower than that in the wild-type WL-3 (Fig. S2). However, no signal of *cpdA* gene was detected in the WL-3Δ*cpdA* (Fig. S2).

### The Effect of CpdA on the Intracellular cAMP Content and Growth

**Fig. 3:**
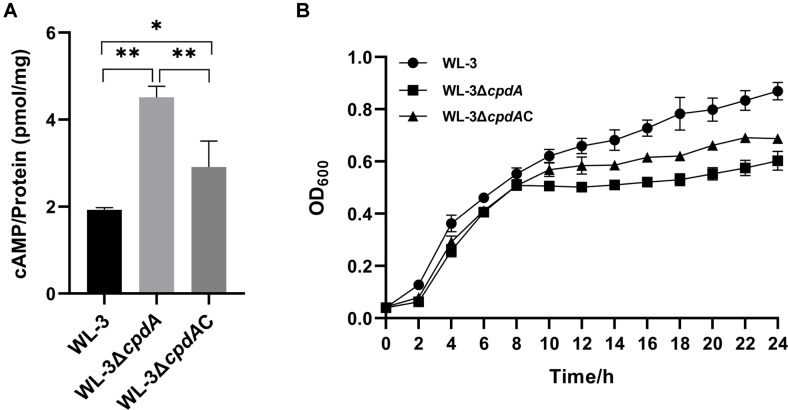
The analysis of intracellular cAMP level and growth of *A. veronii*. (**A**) The functional determination. The WL-3, WL-3Δ*cpdA* and WL-3Δ*cpdA*C were cultured to the OD_600_ of 1.0. The cells were centrifugated, resuspended with sample dilution buffer and then lysed by sonication. After centrifugation, the intracellular cAMP content and total protein concentration in the supernatants were measured with the cAMP Assay Kit and BCA Protein Assay Kit, respectively. The intracellular cAMP level in *A. veronii* was assessed by calculating cAMP concentration per mg of total protein. Data from three independent experiments are presented as mean ± SEM. *: *p* < 0.05, **: *p* < 0.01. (**B**) Growth curve. The overnight cultures of WL-3, WL-3Δ*cpdA* and WL-3Δ*cpdA*C were cultured in the fresh LB medium at the ratio of 1:100. OD_600_ was monitored with an ultraviolet spectrophotometer at the interval of 2 h for 24 h.

The competence ELISA revealed that, compared with wild-type (1.93 ± 0.05 pmol/mg) and WL-3Δ*cpdA*C (2.91 ± 0.60 pmol/mg), WL-3Δ*cpdA* exhibited significantly elevated cAMP levels (4.51 ± 0.26 pmol/mg) compared to representing 2.34-fold and 1.55-fold increases, respectively (Fig. 3A). The cAMP level in WL-3Δ*cpdA*C was partially restored but did not recover to that of wild-type strain, which was consistent with the lower expression of *cpdA* in the WL-3Δ*cpdA*C (Fig. S2). The result indicates that the CpdA protein of *A. veronii* functions as a cyclic AMP PDE, exhibiting cAMP hydrolysis activity *in vivo*. Then, the growth curve in LB medium showed that, compared with WL-3, the *cpdA* deletion inhibited growth rate in the logarithmic and stationary phases (Fig. 3B). The complementary strain WL-3Δ*cpdA*C only partially restored the growth of WL-3Δ*cpdA* from 10 h to 24 h, but not to the level of WL-3 (Fig. 3B). The results indicate that *cpdA* is essential for the growth of *A. veronii*.

### Effects of CpdA on Tolerance to NaCl and pH Stresses

**Fig. 4:**
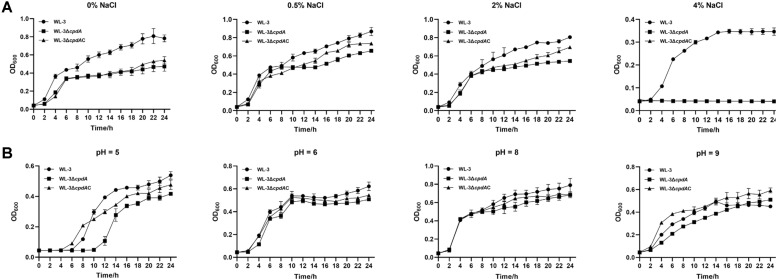
The analysis of tolerance to NaCl and pH. Overnight bacterial cultures were sub-cultured (1% v/v) in LB media with different concentrations of NaCl (0%, 0.5%, 2%, and 4%) or different pH values (5, 6, 8, and 9) and incubated at 37°C. Bacterial growth was monitored by measuring OD_600_ at 2-h intervals using a UV-visible spectrophotometer. Data from three independent experiments are presented as mean ± SEM.

Then, we evaluated the effects of CpdA on the growth under different environment stresses (NaCl and pH). The results showed that WL-3Δ*cpdA* exhibited reduced tolerance to NaCl at concentrations of 0%, 0.5%, 2% and 4%, with negligible growth observed at 4% NaCl (Fig. 4A). Similar growth inhibition of WL-3Δ*cpdA* was observed at different pH stresses (5, 6, 8, and 9) stresses (Fig. 4B). The complementary strain WL-3Δ*cpdA*C partially restored the growth of WL-3Δ*cpdA* at different NaCI (0.5% and 2%) and pH (5, 6, 8 and 9) conditions, but not to the level of WL-3 (Fig. 4B). In contrast, no growth restoration was observed for WL-3Δ*cpdA* under 0% and 4% NaCl stresses (Fig. 4A and 4B). These results demonstrate that the deletion of *cpdA* significantly compromises the adaptation of *A. veronii* to the environment stresses.

### The Effects of CpdA on Biofilm Formation and Bacterial Motility

**Fig. 5:**
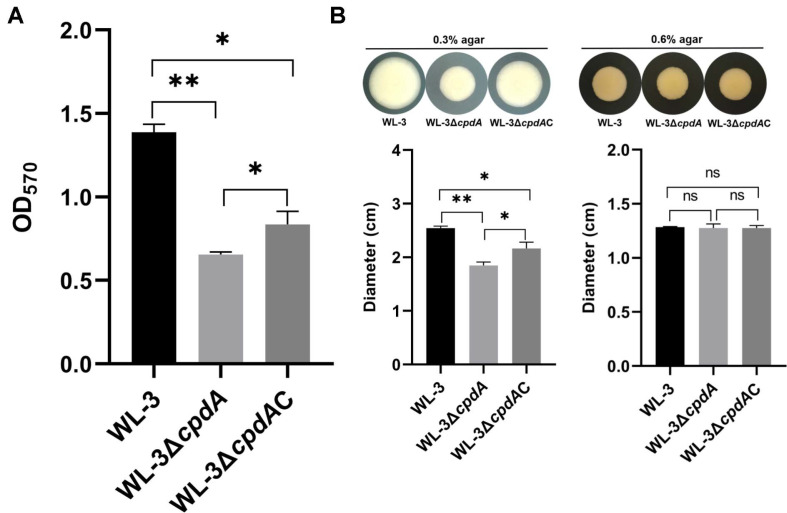
The assay of biofilm formation and motility. (**A**) Biofilm formation. Strains were incubated in 96-well plates at 37°C for 12 h. After washes with PBS, the adherent biofilms were stained with 1% crystal violet and bound dye was solubilized with methanol. Biofilm formation was quantified by measuring the OD_570_. Data from three independent experiments are presented as mean ± SEM. *: *p* < 0.05, **: *p* < 0.01. (**B**) Motility. 1 μl aliquots of WL-3 and WL-3Δ*cpdA* (10^7^ CFU/ml) onto the center of LB plates containing either 0.3% (w/v) agar and 0.6% (w/v) agar, respectively. Motility was quantified by migration diameter after incubation at 37°C for 24 h. Data are the means of three independent experiments and presented as means ± SEM (N = 3). ns: *p* > 0.05, *: *p* < 0.05, **: *p* < 0.01.

Bacterial biofilm formation is regulated with cAMP signal pathway [16]. Therefore, we used the crystal violet staining method to analyze the biofilm formation of WL-3, WL-3Δ*cpdA* and WL-3Δ*cpdA*C. The results showed that after incubation for 12 h, the biofilm formation ability of WL-3Δ*cpdA* (OD_570_: 0.66 ± 0.02) was significantly lower than those of WL-3 (OD_570_: 1.39 ± 0.05) and WL-3Δ*cpdA*C (OD_570_: 0.84 ± 0.08) (Fig. 5A). The results demonstrate that *cpdA* deletion significantly reduces biofilm formation. Combined with the increase in cAMP content observed in the *cpdA* deletion strain WL-3Δ*cpdA* (Fig. 3A), these results indicate that the elevated cAMP level negatively regulates biofilm production.

Based on the above results, we examined the effects of CpdA on motility, which was related to the bacterial biofilm formation and virulence [45, 46] The swimming motility assay showed that the swimming ability of WL-3Δ*cpdA* was significantly attenuated compared to WL-3 and WL-3Δ*cpdA*C (Fig. 5B left). The average swimming diameter of WL-3Δ*cpdA* was 1.85 ± 0.06 cm, which was significantly smaller than those of WL-3 (2.55 ± 0.04 cm) and WL-3Δ*cpdA*C (2.17 ± 0.12 cm) (Fig. 5B left). However, the swarming motility assay showed no significant differences among WL-3, WL-3Δ*cpdA*, and WL-3Δ*cpdA*C (Fig. 5B right). These results indicate a selective role of CpdA in regulation of flagellar-mediated motility.

### The Effects of CpdA on Flagella

**Fig. 6:**
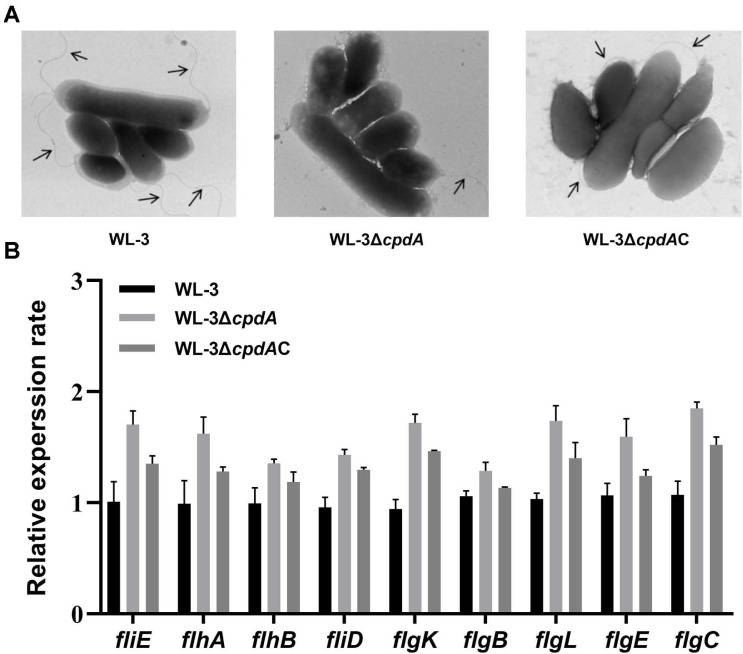
Flagellar morphology observation and flagellar gene expression in *A. veronii* strains. (**A**) Negative-staining TEM. The logarithmic WL-3, WL-3Δ*cpdA* and WL-3Δ*cpdA*C were adsorbed onto formvar/carbon-coated copper grids and stained with 2% phosphotungstic acid. The flagellar morphology was observed with a HT7800 transmission electron microscope. (**B**) qRT-PCR analysis. The WL-3, WL-3Δ*cpdA* and WL-3Δ*cpdA*C were cultured to form biofilm and RNA was extracted by Trizol reagent. The expression level of flagella-related genes (*fliE*, *flhA*, *flhB*, *fliD*, *flgK*, *flgB*, *flgL*, *flgE*, and *flgC*) were quantified by qRT-PCR. Data represent mean ± SD (n=3).

Then, negative staining electron microscopy revealed that, compared with WL-3 and WL-3Δ*cpdA*C, the flagella of WL-3Δ*cpdA* were damaged and shed (Fig. 6A). Also, quantitative analysis of the TEM images revealed that the percentage of WL-3Δ*cpdA* cells with intact flagella (30.8%) was significantly lower than those of WL-3 (91.2%) and WL-3Δ*cpdA*C (57.5%), which was consistent with the observed decrease in swimming motility (Fig. 5B, right). Then, qRT-PCR was used to assess the expression levels of flagella-related genes. Melting curves showed the presence of gene-specific peaks with Tm ranging from 81.21°C to 87.33°C and gel electrophoresis confirmed the single band of the expected size of PCR products (Fig. S1). Amplification efficiency of PCRs reactions varied from 90.73% to 107.53% via the standard curves (Table 2). These results demonstrated that all of primers were specific and efficient. Paradoxically, qRT-PCR showed that, compared with WL-3 and WL-3Δ*cpdA*C, the expression levels of nine flagellar assembly genes (*fliE*, *flhA*, *flhB*, *fliD*, *flgK*, *flgB*, *flgL*, *flgE*, and *flgC*) were all significantly up‐regulated in WL-3Δ*cpdA*, with expression levels increased 1.21 to 1.82 folds and 1.10 to 1.28 folds, respectively (Fig. 6B). These results suggest that *cpdA* deletion disrupts post-transcriptional regulation of flagellar biogenesis, potentially through cAMP-mediated control of flagellar assembly machinery.

### The Effects of CpdA on QS

**Fig. 7:**
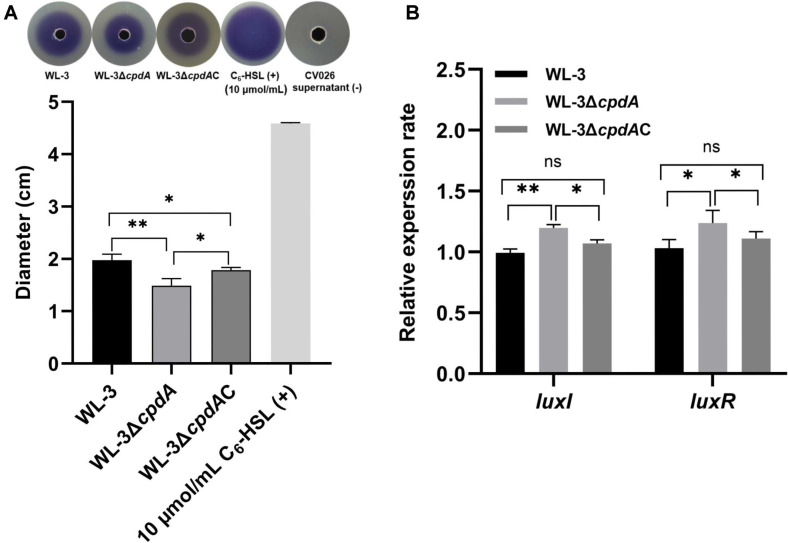
The detection of AHL production and qRT-PCR analysis of QS-related genes. (**A**) The AHL detection. WL-3, WL-3Δ*cpdA* and WL-3Δ*cpdA*C were cultured to OD_600_ of 0.5. After centrifugation, the supernatants were added into the wells in LB agar plates pre-mixed with the biosensor *C. violaceum* CV026. The supernatants of *C. violaceum* CV026 and C_6_-HSL (10 μmol/ml) were used as negative and positive controls, respectively. After incubation at 30°C for 24 h, the diameters of the violet zones were measured to assess the AHL production. * *p* < 0.05, ** *p* < 0.01. (**B**) qRT-PCR analysis. The WL-3, WL-3Δ*cpdA* and WL-3Δ*cpdA*C were cultured to OD_600_ of 0.5 and their RNAs were extracted by Trizol reagent. The expression levels of luxI and luxR were quantified by qRT-PCR. ns: *p* > 0.05, *: *p* < 0.05, **: *p* < 0.01.

AHL detection assays showed that, compared with wild-type WL-3 and WL-3Δ*cpdA*C, WL-3Δ*cpdA* produced significantly smaller purple halos. (Fig. 7A upper). As expected, the negative control (CV026 supernatant) showed no violet halo, whereas the positive control (10 μmol/ml C_6_-HSL) resulted in a clear violet halo (Fig. 7A upper). The average diameter of the violet zones in WL-3Δ*cpdA* (1.49 ± 0.13 cm) was significantly smaller than that in WL-3 (1.98 ± 0.11 cm) and WL-3Δ*cpdA*C (1.78 ± 0.05 cm), respectively, while the purple region generated with the positive control has a diameter of 4.59 ± 0.02 cm (Fig. 7A below). However, qRT-PCR revealed that, compared with WL-3 and WL-3Δ*cpdA*C, the expression levels of QS related genes (*luxI* and *luxR*) were up-regulated in WL-3Δ*cpdA* (Fig. 7B). These results demonstrate that CpdA modulates QS activity in *A. veronii*, likely through post-transcriptional regulation of AHL synthesis.

### The Effects of CpdA on Antibiotic Resistance

**Fig. 8:**
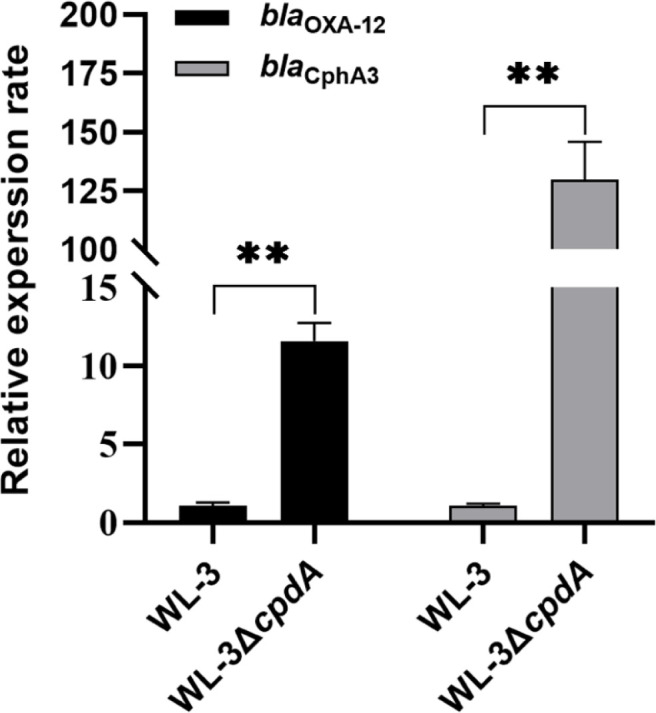
qRT-PCR analysis of antibiotic resistance genes. The WL-3 and WL-3Δ*cpdA* were cultured to OD_600_ of 0.5 and RNA was extracted by Trizol reagent. The expression level of *bla*_CphA3_ and *bla*_OXA-12_ were quantified by qRT-PCR. **: *p* < 0.01.

The disk diffusion assay revealed significant differences in antibiotic susceptibility between WL-3 and WL-3Δ*cpdA* (Table 3). Disk diffusion assays showed that WL-3Δ*cpdA* displayed the markedly increased resistance to β-lactam antibiotics including narrow-spectrum β-lactams (ampicillin, carbenicillin, piperacillin, cephalexin, cefazolin, and cephradine) and extended-spectrum β-lactams (cefuroxime, ceftazidime, ceftriaxone sodium, cefoperazone sodium, imipenem, and meropenem). Furthermore, qRT-PCR analysis revealed that compared to the wild-type WL-3, the expression of two putative β-lactamase genes *bla*_OXA-12_ and *bla*_CphA3_ exhibited in the *A. veronii* WL-3 genome was increased by 11.6- and 129.9-folds in the WL-3Δ*cpdA* mutant, respectively (Fig. 8). Interestingly, the WL-3Δ*cpdA* exhibited increased sensitivity to the aminoglycosides (kanamycin and neomycin), the polypeptide antibiotic (polymyxin B) and the macrolide antibiotic (erythromycin), as evidenced by larger inhibition zones.

In addition, MIC tests confirmed that, compared with WL-3, WL-3Δ*cpdA* exhibited the 2-fold higher MICs for imipenem and meropenem, but 2-fold lower MICs for kanamycin and polymyxin B (Table 4), which were consistent with the results of the disk diffusion assay. These findings indicate that CpdA plays a pleiotropic role in modulating antibiotic resistance pathways.

## Discussion

Bacteria utilize extra- and intracellular signaling molecules to mount appropriate responses to the environmental changes [47]. For pathogenic bacteria, cAMP, as a second messenger, participates in carbon metabolism, biofilm formation, the type III secretion system, and virulence [48]. However, the regulation of cAMP signal pathway in *A. veronii* remained unclear. In this study, the bioinformatics analysis confirmed that *A. veronii* harbored a putative *cpdA* gene with the common conserved sequence motif of Class III PDEs, which shared the similarity of 39.18% - 49.19% with those of other bacteria (Fig. 1). Besides, BLASTp showed that the *cpdA* gene was widely distributed and highly conserved in 90 strains of *A. veronii*, supporting *cpdA* as an indispensable gene of *A. veronii*. For the functional analysis, we successfully constructed a full *cpdA* deletion mutant WL-3Δ*cpdA* (Fig. 2). The intracellular cAMP concentration of WL-3Δ*cpdA* significantly increased by 2.3 folds compared with that of WL-3 (Fig. 3A). These results indicate that the protein encoded by *cpdA* gene exhibits the cAMP PDE activity *in vivo*. Similar cAMP accumulation in *cpdA* mutants also occurs in *E. coli*, *E. piscicida*, *S. marcescens*, *P. aeruginosa* and *Klebsiella pneumoniae* [12, 15, 16, 49, 50], suggesting that Class III PDE-mediated cAMP regulation is a common and conserved strategy in many bacteria. Notably, *cpdA* deletion impaired growth in standard LB medium (Fig. 3B). A comparable growth defect in *Shewanella oneidensis* is attributed to reduced CRP-dependent cytochrome bd and cbb3 oxidase expression [51]. These results support that the regulation of cAMP plays essential roles on bacterial reproduction.

The dynamic balance of cAMP is necessary for a variety of bacterial functions including stress tolerance, motility, biofilm formation, and pathogenicity [52]. In this study, we found that, compared with the wild-type strain, the *cpdA* defect reduced the tolerance of *A. veronii* to NaCl (from 0% to 4%) (Fig. 4 A). The increase of cAMP in pathogenic *M. tuberculosis* and *Anabaena* sp. PCC 7120 is triggered in response to NaCl stress [53, 54]. The reduced NaCl tolerance observed in WL-3Δ*cpdA* might result from a dysregulation of the normal cAMP-mediated response. Furthermore, WL-3Δ*cpdA* showed reduced tolerance to both acidic and alkaline stresses (Fig. 4 A). Interestingly, although cAMP concentration was elevated in WL-3Δ*cpdA*, we found that the expression of *crp* was down-regulated by 2.11-fold relative to that of WL-3 (Fig. S3). This contrasts with the established *E. coli* model, where the absence of cAMP-CRP enhances acid resistance and alkaline condition stimulates high cAMP production to maintain survival [55, 56]. Collectively, our results indicate that, in *A. veronii*, the responses to acid and alkaline stresses are mediated by the cAMP-CRP signaling pathway.

Also, we found that the deletion of *cpdA* inhibited the biofilm-forming ability and swimming motility (Fig. 5). The result was consistent with *E. piscicida*, *S. marcescens* and *P. aeruginosa* [15, 16, 57]. cAMP accumulation inhibits biofilm formation through suppression of irreversible attachment mediated by fimbriae and flagella [16, 57]. Flagellar motility and QS required for biofilm formation and virulence are regulated by the cAMP signal pathway [58, 59]. TEM showed that the flagellar morphology was damaged and shed (Fig. 6A) and the production of AHL was attenuated in WL-3Δ*cpdA* (Fig. 7A), which supported the decreased swimming motility and biofilm formation. The role of PDE in *A. veronii* is similar with cyclic-di-GMP PDE in *V. parahaemolyticus*, which regulates the expression of polar and lateral flagellar genes and the biofilm-related gene cpsA [60]. However, qRT-PCR showed that the transcriptional levels of nine flagella-related genes and QS-related genes (*luxI* and *luxR*) were upregulated in the WL-3Δ*cpdA* (Figs. 6B and 7B), which was contradictory with the reduced phenotypes in the flagella-mediated motility and AHL production. Although our results indicate that CpdA might be involved in post-transcriptional regulation, this hypothesis requires future validation through direct protein-level analysis. Also, the synthesis and assembly of flagella and regulation of QS are complex and involve intricate networks of genes, proteins, and signaling molecules [61, 62]. To further investigate the role of CpdA, the future studies should employ transcriptomic and proteomic analyses to elucidate its regulatory networks and functional dynamics.

Antibiotic-resistant bacteria (ARB) pose a serious threat to the global public health, and there is an urgent need to develop novel antibacterial agents targeting new proteins to combat ARB [63, 64]. In our study, the results showed that WL-3Δ*cpdA* exhibited markedly higher resistance to β-lactam antibiotics (Table 3) and MICs for imipenem and meropenem were 2-fold higher than those of WL-3 (Table 4), which was in agreement with loss of cyclic-di-AMP PDE in *Staphylococcus aureus* [65]. Also, our discovery that flagellar impairment coincided with increased antibiotic resistance in *A. veronii* WL-3Δ*cpdA* was supported by the mechanistic evidence from *Salmonella*, where flagellin deficiency served a key driver of multidrug resistance through biofilm adaptation and efflux pump activation [66]. While this parallel maybe suggests a conserved strategy where flagellin loss triggers antibiotic-resistant adaptations, the precise relationship between flagellar function and drug resistance in *A. veronii* requires further investigation. In addition, the isolated *A. veronii* strains harbor the different β-lactamase genes such as *bla*_CphA3_, *bla*_OXA-12_, *bla*_FOX-2_, and *bla*_VEB-28_, resulting in hydrolyzing extended-spectrum β-lactam antibiotics and conferring resistance [67, 68]. Also, we found that the observed upregulation of the putative β-lactamase genes *bla*_CphA3_ and *bla*_OXA-12_ in WL-3Δ*cpdA* (Fig. 8) was consistent with the enhanced β-lactam resistance in the mutant, suggesting that increased β-lactamase activity likely contributed to this phenotype. Interestingly, the deletion of *cpdA* increased the sensitivity to four drugs (Table 3) and MICs for kanamycin and polymyxin B were 2-fold lower than those of WL-3 (Table 4). Inhibition of cAMP activity promotes the synthesis and addition of 4-amino-4-deoxy-l-arabinose (l-Ara4N) to lipopolysaccharide (LPS) and increased bacterial resistance to polymyxin B in *E. coli* [69]. Also, the cAMP-CRP involves in regulation of the membrane impermeability and efflux pump, which are directly related to antibiotic resistance [70].

Pharmacological inhibitor of CpdA combined with kanamycin or polymyxin B might lower the required antibiotic dose and circumvent antibiotic resistance. Similarly, targeting the PDE Rv1339 in mycobacteria has been proposed as a strategy to synergize with cell-wall-targeting antimicrobials [17]. Therefore, inhibition of CpdA in *A. veronii* would not only attenuate virulence by impairing biofilm formation, motility, and stress adaptation, but also increase susceptibility to conventional antibiotics. Our study establishes CpdA as a promising therapeutic target with a dual mechanism of action.

## Conclusion

In this study, the bioinformatic and functional analysis confirmed for the first time that *A. veronii* produces an active class III cAMP PDE, named CpdA. The deletion of *cpdA* significantly impaired the bacterial growth on normal LB medium, reduced the tolerance to NaCl and pH stress, inhibited the biofilm formation and attenuated swimming motility. Mechanistically, the loss of *cpdA* disrupted flagellar structural integrity and diminished AHL production in the QS system, despite paradoxically upregulating the expression of nine flagella-related genes and two QS-related genes. Interestingly, WL-3Δ*cpdA* displayed higher resistance to β-lactam antibiotics with up-regulation of *bla*_CphA3_ and *bla*_OXA-12_, but enhanced sensitivity to kanamycin, neomycin, polymyxin B, and erythromycin. Notably, genetic complementation in strain WL-3Δ*cpdA*C partially or fully recovered most of the phenotypes lost in WL-3Δ*cpdA*. These results established CpdA protein as a vital regulator of multiple phenotypes of *A. veronii* and positioned CpdA protein as a potential target for therapeutic strategies.

## Supplemental Materials

Supplementary data for this paper are available on-line only at http://jmb.or.kr.



## Figures and Tables

**Table 1:** Primers used for the deletion mutant and complementary strain.

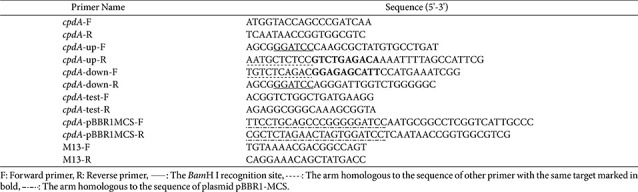

**Table 2:** Primers for qRT-PCR.

Primer Name	Sequence (5'-3')	Size (bp)	Efficiency (%)
*cpdA*-RT-F	GCCAATGAACACCCCTACGA	197	108.24
*cpdA*-RT-R	AGCTGCTTGGATTCGCTGAT		
*fliE*-RT-F	TAACCTCCGACTTTGGTCAGC	144	90.80
*fliE*-RT-R	ATTTTTGGCCCGCGATCATC		
*flhA*-RT-F	ACCCATCTGAGCCAGATCCT	181	102.24
*flhA*-RT-R	GCACGCCTTCATTGAGCAAA		
*flhB*-RT-F	TGATCATCCTCTGCTGTGCC	161	91.83
*flhB*-RT-R	CAACTGACGGATCTTGCCCT		
*fliD*-RT-F	TCCCCTGCCTTGACATCAAC	191	98.60
*fliD*-RT-R	CCGGTGATGAAGGTGGAGAG		
*flgK*-RT-F	TGACCATGCCACTCAACTCC	181	91.14
*flgK*-RT-R	TATCATGCCTCCCTGACCCA		
*flgB*-RT-F	ACCAAAACCCTGATGCGACT	156	90.73
*flgB*-RT-R	GCATTCGGTGTGCATCAACA		
*flgL*-RT-F	ATCAGCTTCCGACACCTTGG	195	99.43
*flgL*-RT-R	CCCGCTATTACCGCTGACAA		
*flgE*-RT-F	CACGGGTATAGGTGCGATCC	169	107.21
*flgE*-RT-R	AATGCCAAGACTCAGGTCGG		
*flgC*-RT-F	TCTTCTTGGCCGAATCTGCA	145	105.44
*flgC*-RT-R	GAGTACAACCCCAACACCCC		
*luxR*-RT-F	AACGGCATCTCCTTCCCTCT	184	108.20
*luxR*-RT-R	TACCCGCACTGTCCATCATG		
*luxI*-RT-F	CCTGATCGCCACGCTCTTAT	110	93.19
*luxI*-RT-R	GCACCTCGTCAAACTCCTGA		
*bla*_OXA-12_-RT-F	TGCAGTACACCGCCAATATC	176	92.13
*bla*_OXA-12_-RT-R	GGTATGGACGAAGATCAGCTGT		
*bla*_CphA3_-RT-F	TGCACAAGCTGATCAAACGG	158	107.53
*bla*_CphA3_-RT-R	CCAGTCGCTCTTCATCAGAT		
*crp*-RT-F	ACGATGAGGGCAAAGAGATGA	160	94.18
*crp*-RT-R	TGGCGGAACTTCTTGTAGGA		
16S rRNA-RT-F	TTCGATGCAACGCGAAGAAC	197	109.03
16S rRNA-RT-R	TCCCTTGAGTTCCCACCATTAC		

**Table 3:** The disk diffusion assay.

Number	Antibiotic	Content	Average diameter/cm		P
			WL-3	WL-3Δ*cpdA*	
1^a^	Polymyxin B	300 IU	1.35 ± 0.04	1.68 ± 0.08	0.0035^**^
2^a^	Kanamycin	30 μg	0.99 ± 0.01	1.45 ± 0.02	< 0.0001^**^
3^a^	Neomycin	30 μg	1.36 ± 0.04	1.78 ± 0.22	0.0285^*^
4^a^	Erythromycin	15 μg	1.10 ± 0.02	1.36 ± 0.05	0.0008^**^
5^b^	Imipenem	10 μg	1.25 ± 0.04	1.02 ± 0.04	0.0025^**^
6^b^	Meropenem	10 μg	2.16 ± 0.03	1.51 ± 0.02	< 0.0001^**^
7^b^	Ampicillin	10 μg	1.75 ± 0.17	0.70 ± 0.00	0.0002^**^
8^b^	Carbenicillin	100 μg	2.08 ± 0.01	0.70 ± 0.00	< 0.0001^**^
9^b^	Piperacillin	100 μg	2.84 ± 0.12	1.11 ± 0.06	< 0.0001^**^
10^b^	Cephalexin	30 μg	2.00 ± 0.08	1.77 ± 0.11	0.0485^*^
11^b^	Cefazolin	30 μg	2.42 ± 0.11	2.15 ± 0.06	0.0192^*^
12^b^	Cephradine	30 μg	2.28 ± 0.06	2.02 ± 0.03	0.0030^**^
13^b^	Cefuroxim	30 μg	2.86 ± 0.12	2.49 ± 0.03	0.0061^**^
14^b^	Ceftazidime	30 μg	2.35 ± 0.05	1.78 ± 0.04	< 0.0001^**^
15^b^	Ceftriaxone Sodium	30 μg	3.89 ± 0.16	3.06 ± 0.04	0.0178^*^
16^b^	Cefoperazone sodium	75 μg	3.81 ± 0.11	2.24 ± 0.01	< 0.0001^**^

^a^: decreased resistance; ^b^: increased resistance; ^*^: *p* < 0.05; ^**^: *p* < 0.01.

**Table 4:** Minimum Inhibitory Concentration (μg/mL).

	Imipenem	Meropenem	Kanamycin	Polymyxin B
WL-3	64	32	256	128
WL-3Δ*cpdA*	128	64	128	64
