# Supplementary material for: The Cyclic Adenosine Monophosphate Phosphodiesterase CpdA Modulates cAMP Homeostasis, Stress Adaptability, Biofilm Formation, Motility, Quorum Sensing and Antibiotic Resistance of Aeromonas veronii
Source: J Microbiol Biotechnol. 2026 Apr 15;36:e2511034. doi: 10.4014/jmb.2511.11034 (PMC13087886; doi:10.4014/jmb.2511.11034)
Supplement: Supplementary file 1 [file jmb-36-e2511034-supple.pdf]

**J. Microbiol. Biotechnol. 2026. 36: e2511034**
https://doi.org/10.4014/jmb.2511.11034

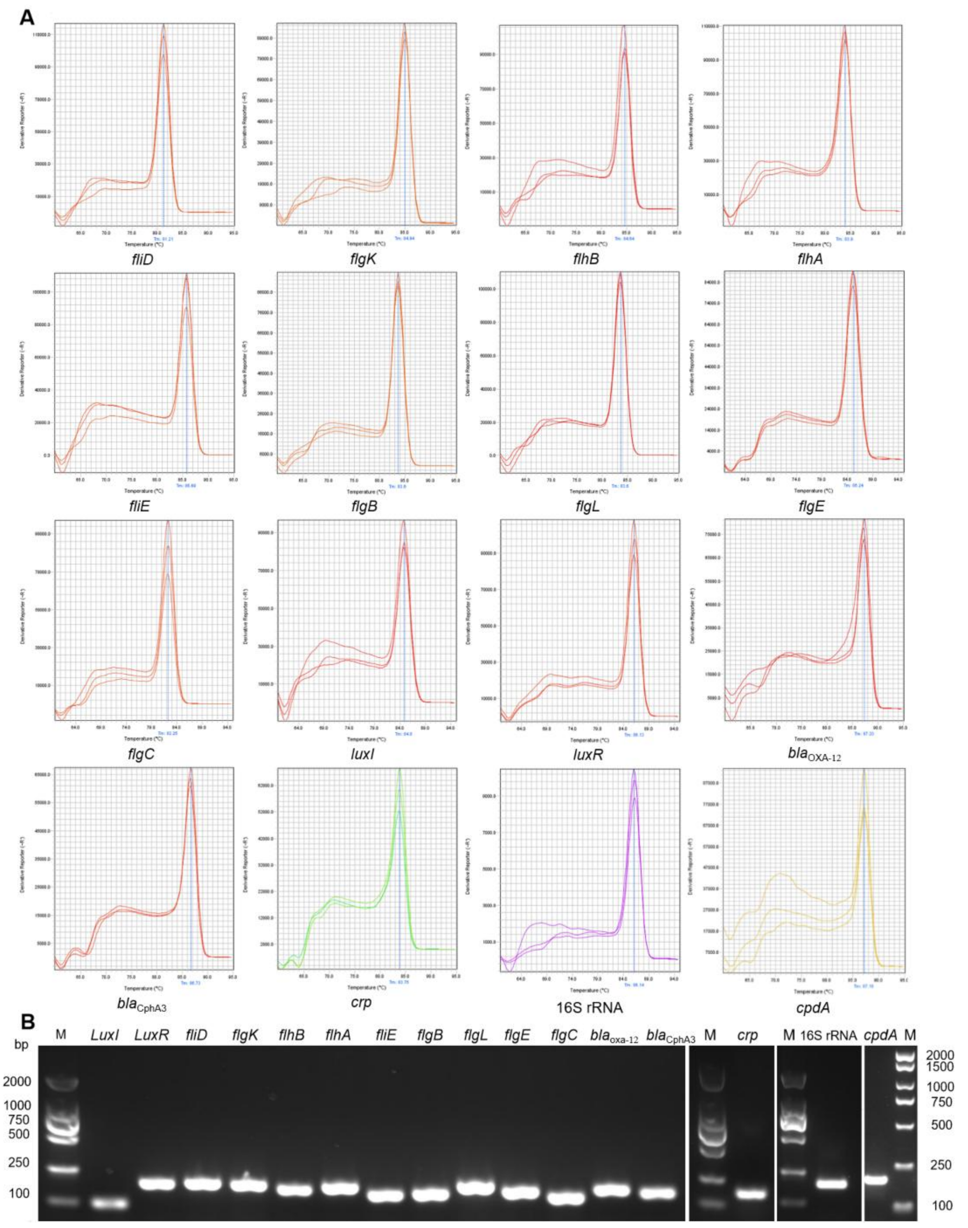


**Fig. S1. The verification of the specificity of primers in qRT-PCR. (A**) Analysis of melting curves. **(B)** Agarose gel electrophoresis analysis.

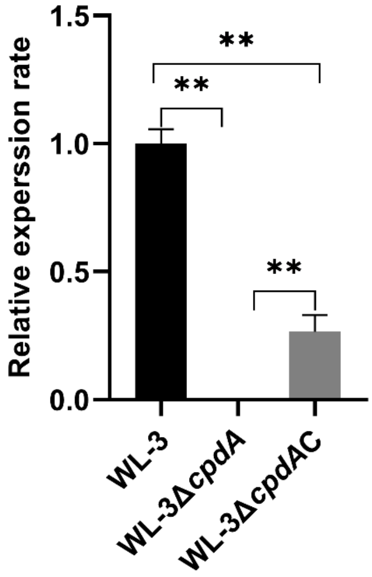


**Fig. S2. qRT-PCR analysis of *cpdA* gene**. The WL-3, WL-3Δ*cpdA* and WL-3Δ*cpdA*C were cultured to $OD_{600}$ of 0.5 and RNA was extracted by Trizol reagent. The expression level of *cpdA* was quantified by qRT-PCR. ** $p < 0.01$.

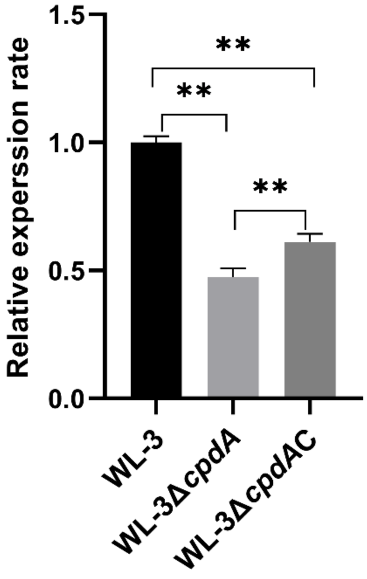


**Fig. S3. qRT-PCR analysis of *crp* gene**. The WL-3, WL-3Δ*cpdA* and WL-3Δ*cpdA*C were cultured to $OD_{600}$ of 0.5 and RNA was extracted by Trizol reagent. The expression level of *crp* was quantified by qRT-PCR. ** $p < 0.01$.
